# Separation Properties of Plasmid DNA Using a Two-Stage Particle Adsorption-Microfiltration Process

**DOI:** 10.3390/membranes13020168

**Published:** 2023-01-29

**Authors:** Nobuyuki Katagiri, Daisuke Shimokawa, Takayuki Suzuki, Masahito Kousai, Eiji Iritani

**Affiliations:** 1 Department of Environmental Technology, Meijo University, 1-501 Shiogamaguchi, Tempaku-ku, Nagoya 468-8502, Japan; 2 Department of Chemical Engineering, Nagoya University, Furo-cho, Chikusa-ku, Nagoya 464-8603, Japan

**Keywords:** microfiltration, adsorption, desorption, plasmid DNA, affinity, ligand

## Abstract

Plasmid DNA is used as a vector for gene therapy and DNA vaccination; therefore, the establishment of a mass production method is required. Membrane filtration is widely employed as a separation method suitable for the mass production of plasmid DNA. Furthermore, the separation of plasmid DNA using microfiltration and ultrafiltration membranes is being investigated. Because plasmid DNA has a circular structure, it undergoes significant deformation during filtration and easily permeates the membrane, hindering the selection of separation membranes based on molecular weight. In this study, we applied affinity microfiltration to plasmid DNA purification. *α*-Fe_2_O_3_ with an isoelectric point of approximately 8 and a particle size of 0.5 μm was selected as the ligand for two-stage affinity microfiltration of plasmid DNA. In the first stage of microfiltration, the experiment was conducted at a pH of 5, and a cake of *α*-Fe_2_O_3_ with bound plasmid DNA was obtained. Next, liquid permeation (pH 9 and 10) through the cake was performed to elute the bound plasmid DNA. Plasmid DNA was eluted during the early phase of liquid permeation at pH 10. Furthermore, agarose gel analysis confirmed the usefulness of the two-stage affinity microfiltration method with adsorption and desorption for plasmid DNA purification.

## 1. Introduction

Recently, gene therapy and DNA vaccines have been actively developed for the treatment of various diseases [1]. Gene therapy requires a vector that acts as a carrier for gene replacement, and plasmids are used as non-viral vectors [2,3]. Plasmid DNA is an extranuclear gene that exists in bacteria such as *Escherichia coli* and replicates independently of chromosomal DNA. To use plasmid DNA in gene therapy, it is necessary to mass-produce pharmaceutical-grade plasmid DNA and develop industrial-scale isolation and purification methods [4,5]. Plasmid DNA purification starts with the process of extracting plasmid DNA from the inside of the bacterial cells, followed by lysis through the addition of chemicals, separation of the bacterial mass, and chromatography [6,7]. The use of hazardous substances that affect the human body is preferably avoided, and a safe, scalable, and cost-effective purification process for plasmid DNA needs to be developed.

Membrane processes have immense potential for large-scale plasmid purification. Several studies [8,9,10,11,12,13,14,15] have demonstrated that membrane-based processes are effective for the purification of plasmid DNA. Microfiltration membranes are mainly used to remove contaminants such as chromosomal DNA, proteins, and aggregates of bacteria because capturing nanosized plasmid DNA is difficult [11,14]. In contrast, ultrafiltration membranes that can capture nanosized particles are used to capture and purify or concentrate plasmid DNA [14]. However, as plasmid DNA has a circular structure and is significantly deformed during filtration, it may permeate the membrane, depending on the filtration conditions, even when an ultrafiltration membrane is used. Therefore, although the selection of separation membranes based on molecular weight is difficult, several studies have been conducted on the physical mechanisms governing DNA transmission and the effects of membrane pore size and operating conditions on the DNA sieving coefficient [9,10,12].

Affinity membrane filtration, in which a large ligand is used to selectively bind the desired materials in solution and is retained by a semipermeable membrane, is a promising purification technique for biopolymers [16,17]. Using this method, plasmid DNA can be adsorbed onto submicron-sized ligands and captured using microfiltration membranes. Since the performance of affinity filtration is significantly influenced by the specific binding interactions between the targeted material and the ligand, various types of ligands have been applied to attain a higher level of separation efficiency for biopolymers. Affinity substances for plasmid DNA include metal ions, metal oxides, peptides, and proteins [18,19,20,21]. In general, these ligands are used as a fixed layer; however, in this study, we investigated a membrane filtration method in which metal oxide particles of a size that can be captured using a microfiltration membrane are added as ligands to a solution containing plasmid DNA.

*E. coli* is often used for the production of plasmid DNA, and several extraction methods have been investigated [22,23]. Cell disruption for plasmid DNA extraction should be performed to minimize damage to plasmid DNA and genomic DNA. Alkaline lysis is the most commonly used method for cell disruption; however, it has known limitations, including low plasmid DNA recovery and a time-consuming process. Haberl et al. showed that electroextraction is a swifter alternative to alkaline lysis for the extraction of plasmid DNA [22]. Padilla-Zamudio et al. showed that cell disruption in a bead mill was more efficient in releasing plasmid DNA than alkaline lysis [23]. High pressure is also effective for cell disruption, and it is known that metabolites such as nucleic acids can be extracted from *E. coli* cells at pressures above 600 kPa [24]. Each extraction method has advantages and disadvantages; therefore, to establish a highly efficient purification method for plasmid DNA, an examination of the extraction of plasmid DNA, including its separation properties after cell disruption, is necessary.

In the present study, the application of affinity microfiltration to plasmid DNA purification and the search for ligands was examined. In addition to the selectivity behaviors in the binding process of plasmid DNA to the ligand and the elution process of the bound plasmid DNA, the membrane filtration behaviors of plasmid and ligand were also investigated in this system. Furthermore, we investigated the cell disruption method for plasmid DNA extraction and the membrane filtration properties of the disrupted suspension. The results of this study demonstrated the effectiveness of a two-stage microfiltration process, in which both the adsorption and desorption of plasmid DNA to large ligands exhibit immense potential for plasmid DNA purification.

## 2. Materials and Methods

### 2.1. Materials

A 3.0 kb plasmid DNA pBluescript II SK(+) was obtained from Stratagene Corp., San Diego, CA, USA. *Escherichia coli* DH5*α* (Nippon Gene Co. Ltd., Tokyo, Japan) was used as the host for the plasmid and grown at 303 K on an LB medium supplemented with the ampicillin antibiotic. The test solution was prepared by the following three steps: alkaline lysis of *E. coli* containing plasmid DNA, the addition of CaCl_2_ for the removal of high molecular weight RNA [25], and the addition of ethanol for the concentration of nucleic acid. The plasmid DNA-containing sediment was dissolved in 10 mM Tris-HCl buffer (pH 5), and this solution, free of impurities such as proteins, was used for a two-stage affinity microfiltration experiment. The ligand employed in the experiments was *α*-Fe_2_O_3_ (particle size: 0.5 μm) provided by the Kojundo Chemical Lab. Co. Ltd., Saitama, Japan. A microelectrophoresis Mark II apparatus (Rank Brothers Ltd., Cambridge, UK) was used to determine the zeta potential of *α*-Fe_2_O_3_ particles.

### 2.2. Adsorption and Desorption Experiments

Plasmid DNA solutions of different concentrations were prepared and added to *α*-Fe_2_O_3_ slurries with known concentrations (0.1–80 mg/mL) to measure the adsorption properties. The solvents used for the solutions and slurries were pH 5–7 10 mM Tris-HCl buffer. An amount of 1 mL of each solution was maintained at a constant temperature of 298 K for 1 h, which was confirmed to be sufficient to achieve a quasi-steady state in the preliminary test. The desorption of the plasmid DNA adsorbed onto the particles was performed by changing the pH of the solution and allowing it to stand for 1 h. The amounts of adsorbed or desorbed plasmid DNA were determined from the concentrations of plasmid DNA in the solutions before and after the experiments using a spectrophotometer (UV-1800, Shimadzu Corp., Kyoto, Japan). The plasmid DNA used in the adsorption/desorption experiments was purified using a Qiagen plasmid midi kit.

### 2.3. Two-Stage Affinity Microfiltration Experiments

An unstirred batch filtration cell with an effective membrane area of 19.6 cm^2^ was utilized in this study. Microfiltration experiments were performed in the dead-end filtration mode under constant pressure by applying compressed nitrogen gas [26]. The filtrate was collected in a reservoir placed on an electronic balance (Shimadzu Corp., Kyoto, Japan) connected to a personal computer to collect and record mass versus time data. The weights were converted to volumes using density correlations. A mixed cellulose ester microfiltration membrane (Advantec Toyo Co. Ltd., Tokyo, Japan) with a nominal pore size of 0.1 μm was employed in the experiments. In the first stage of microfiltration, the experiment was conducted under the condition of pH 5 using test solutions containing ligands (160 mL, the mass fraction of the solids *s* = 0.016), and a cake of *α*-Fe_2_O_3_ with plasmid bound was obtained. In the second stage of microfiltration, 10 mL of 1 M Tris-HCl buffer (pH 9) was added to the top of the cake and allowed to permeate after standing for 1 h. Next, the permeation of 2 M Tris-HCl buffer (pH 10, 50 mL) through the cake was performed to elute the bound plasmid DNA. The concentration of plasmid DNA in the permeate was measured at 260 nm using a spectrophotometer (UV-1800, Shimadzu Corp., Kyoto, Japan). The quality of the plasmid DNA was confirmed using agarose gel electrophoresis. A permeate of 5 μL was mixed with 1 μL of 6×loading buffer (0.25% bromophenol blue, 0.25% xylenecyanol, and 5 mM EDTA in 30% glycerol) and subjected to electrophoresis. Electrophoresis was performed in 0.6% (*w*/*v*) agarose (Nippon Gene Co. Ltd., Tokyo, Japan) containing ethidium bromide for 1 h at 100 V for using a submarine electrophoresis system (Nihon Eido Co.Ltd., Tokyo, Japan). Gels were placed on a UV table (Atto Corp., Tokyo, Japan), and photographs were taken with Polaroid (Funakoshi Co. Ltd., Tokyo, Japan). OneSTEP Marker 1 (λ/Hind III digest, Nippon Gene Co. Ltd., Tokyo, Japan) was used as a molecular marker. The two-stage affinity microfiltration experiments were performed more than three times to ensure the reproducibility of the results.

### 2.4. Cell Disruption Experiments

Three mechanical cell disruption methods were investigated to extract plasmid DNA from *E. coli* cells. The *E. coli* cells, after cultivation, were collected using centrifugation (3000 rpm, 15 min) and suspended in pure water to prepare a suspension (3.0 × 10^8^ cell/mL). 10 mL of *E. coli* suspension was disrupted with an ultrasonic homogenizer (UP-200S, Dr. Hielscher GmbH, Stuttgart, Germany) at an operating frequency of 24 kHz and a nominal load power output of 200 W for 60 s. Cell disruption using a bead mill was performed by setting a 2 mL tube (zirconia beads of 1 mm diameter, 60 beads, Sarstedt Inc., Newton, MA, USA) in a Delta Mixer (Se-08, Taitec Corp., Tokyo, Japan) and shaking for 30 min at 3000 rpm. An attempt was made to extract the plasmid DNA from the cells using electroporation. The condition was as follows: 0.2 cm-gap sterile electroporation cuvette, pulse number 10, voltage 500 V, pulse length 100 ms, and interval 0.1 s using the Gene Pulser Xcell Electroporation System (Bio-Rad Laboratories, Inc., Hercules, CA, USA). Photomicrographs of *E. coli* after disruption were obtained using a digital photomicroscope (BA210EINT, Shimadzu Rika Corp., Tokyo, Japan). A suspension of disrupted *E. coli* cells was subjected to affinity filtration after removing solids with constant pressure microfiltration (*p* = 49 kPa, mixed cellulose ester membrane with 0.1 μm).

## 3. Results and Discussion

### 3.1. Adsorption and Desorption Properties of Plasmid DNA

**Figure 1:**
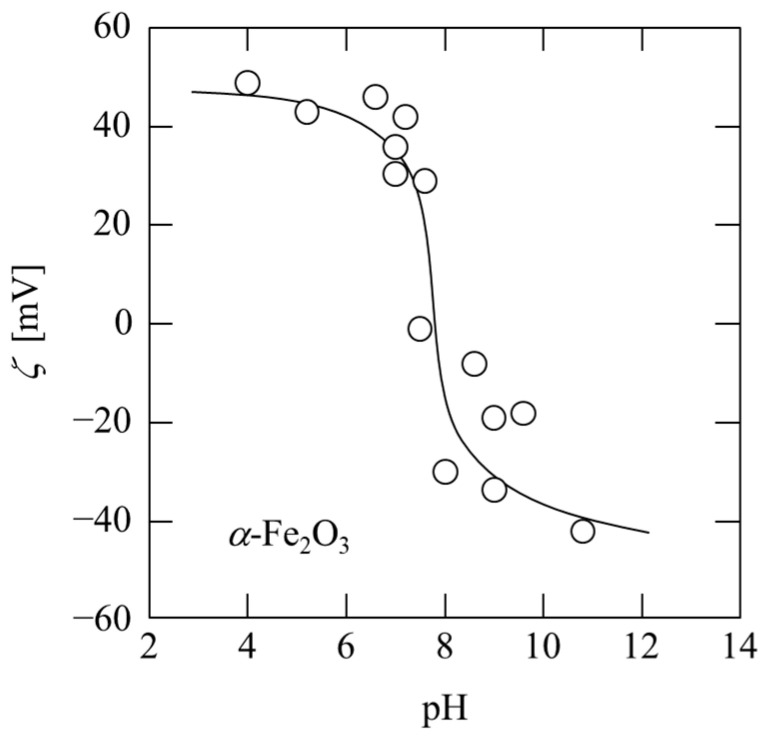
pH dependence of zeta potential of *α*-Fe_2_O_3_ particles.

Figure 1 shows the pH dependence of the zeta potential of *α*-Fe_2_O_3_ particles used as ligands. The isoelectric point is approximately pH 8, and it is positively charged at a pH lower than eight and negatively charged at a pH above eight. Since plasmid DNA is a polyanion, it is expected to be adsorbed on the surface of positively charged *α*-Fe_2_O_3_ by setting the pH below seven. In contrast, in a solution environment with a pH greater than nine, an electrostatic repulsive force acts between *α*-Fe_2_O_3_ and plasmid DNA.

**Figure 2:**
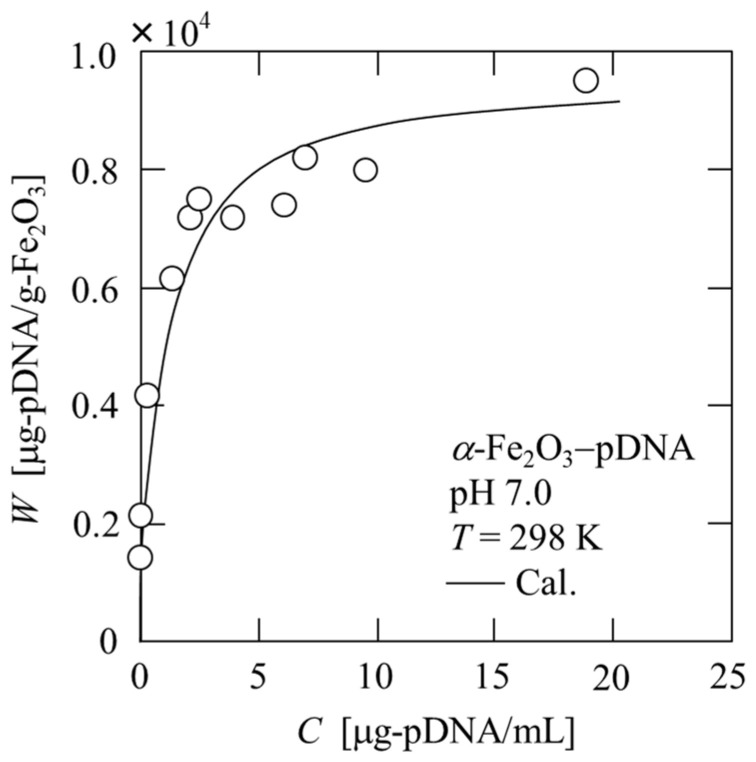
Property of adsorption of plasmid DNA onto *α*-Fe_2_O_3_.

The adsorption isotherms of plasmid DNA onto *α*-Fe_2_O_3_ were obtained through batch adsorption experiments, and the results at pH 7 are shown in Figure 2. The experimental data were approximated using the Langmuir adsorption isotherm equation, represented by

(1) $$W=\frac{aW_{s}C}{1+aC},$$

where *W* is the amount of plasmid DNA adsorbed, *a* is the Langmuir adsorption constant, *W*_s_ is the maximum adsorption capacity of *α*-Fe_2_O_3_ for plasmid DNA, and *C* is the equilibrium concentration of plasmid DNA in the solution. The solid line in the figure represents the calculated value based on Equation (1). This result is consistent with the findings of Liu et al., in which the DNA adsorption behavior of modified magnetic nanoparticles follows the Langmuir isotherm model [27]. As can be seen from the figure, the amount of adsorption is large, even at extremely low concentrations, and the affinity of plasmid DNA for *α*-Fe_2_O_3_ is extremely high. However, the maximum adsorption amount of plasmid DNA on the modified magnetic nanoparticles used by Liu et al. was approximately 10 times larger than that on the iron oxide particles we used. It is expected that the adsorption amount of plasmid DNA can be increased by modifying the surface of the iron oxide particles.

**Figure 3:**
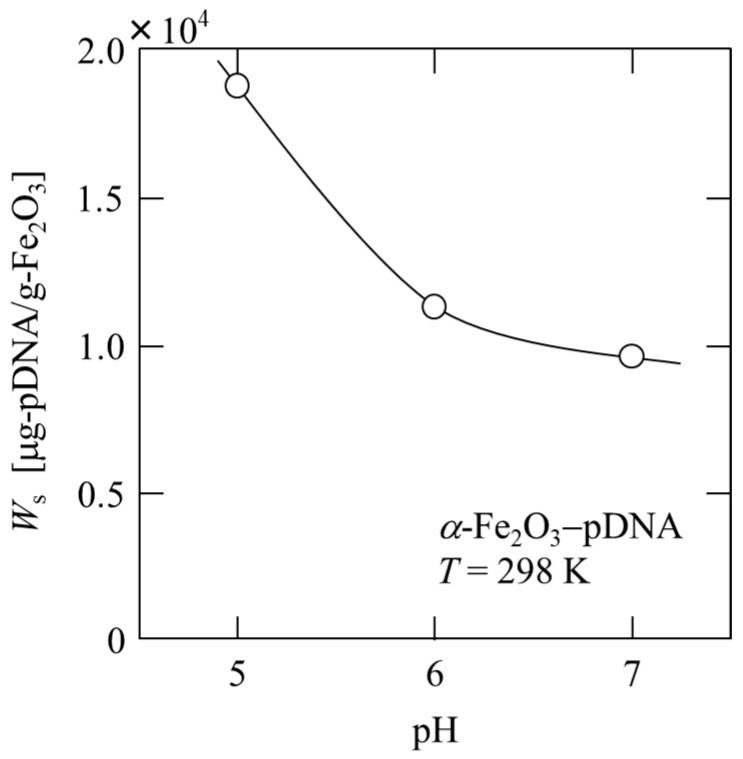
Dependence of adsorption capacity of plasmid DNA on the pH of the solution.

In Figure 3, the maximum adsorption capacity *W*_s_ is plotted against the pH of the solution. The amount of plasmid DNA adsorbed is strongly dependent on pH and decreases with increasing pH at pH 5–7. The *W*_s_ at pH 5 was approximately twice that at pH 7. By lowering the pH, more plasmid DNA can be adsorbed; however, if it is extremely low, plasmid DNA may deteriorate. Therefore, the first stage of microfiltration involving the binding of plasmid DNA was performed at pH 5.

**Figure 4:**
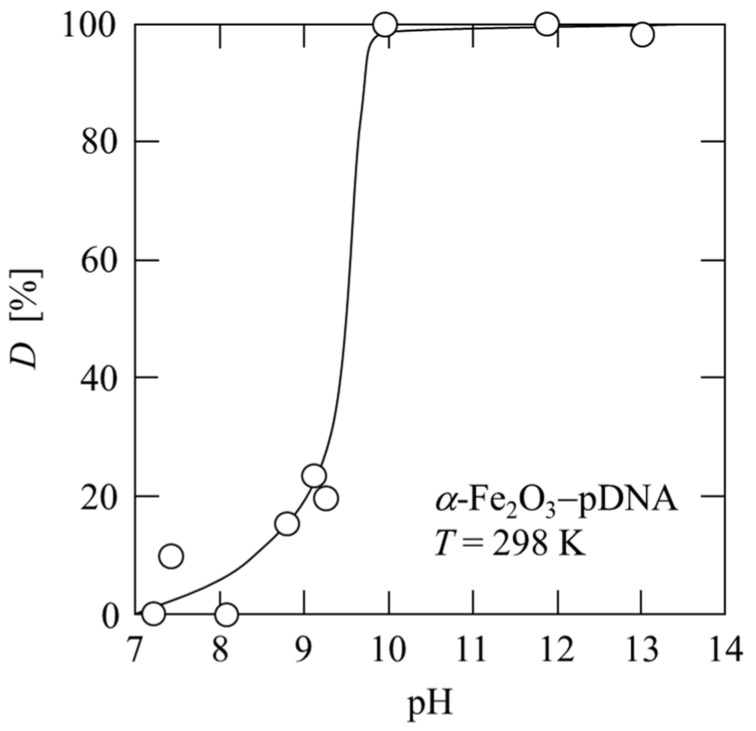
Desorption property of plasmid DNA corresponding to the pH change of the solution.

Figure 4 shows the effects of pH on the desorption of plasmid DNA from *α*-Fe_2_O_3_. After the plasmid was adsorbed onto *α*-Fe_2_O_3_, the pH of the solution was gradually increased, and the desorption efficiency *D* of the plasmid DNA was determined by measuring the amount of desorbed plasmid DNA that migrated into the solution. The plasmid DNA was desorbed at a pH above the isoelectric point of *α*-Fe_2_O_3_, and the desorption efficiency *D* was approximately 100% above pH 10. At pH 10, the plasmid DNA was desorbed from *α*-Fe_2_O_3_ particles (0.125 mg/mL) and recovered as a solution with a concentration of 1.6 μg/mL. Impurities can be separated using adsorption filtration of the plasmid DNA, and subsequently, the plasmid DNA can be recovered using desorption filtration. Therefore, *α*-Fe_2_O_3_ particles are determined to be suitable as a ligand. The second stage of microfiltration involving the desorption of plasmid DNA was performed using liquid permeation with a stepwise increase in pH.

### 3.2. Two-Stage Affinity Microfiltration Properties of Plasmid DNA

**Figure 5:**
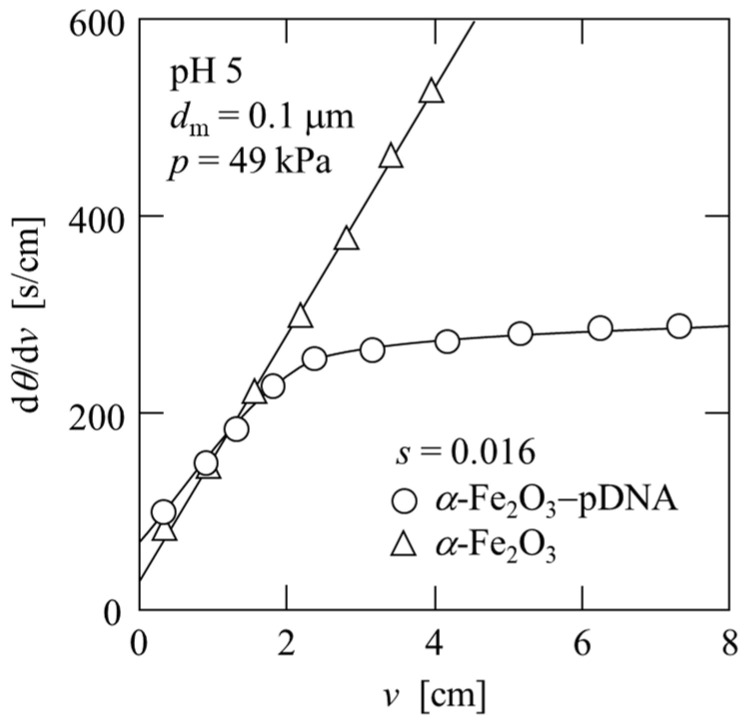
Relation between reciprocal filtration rate and filtrate volume per unit membrane area in adsorption-filtration.

**Figure 6:**
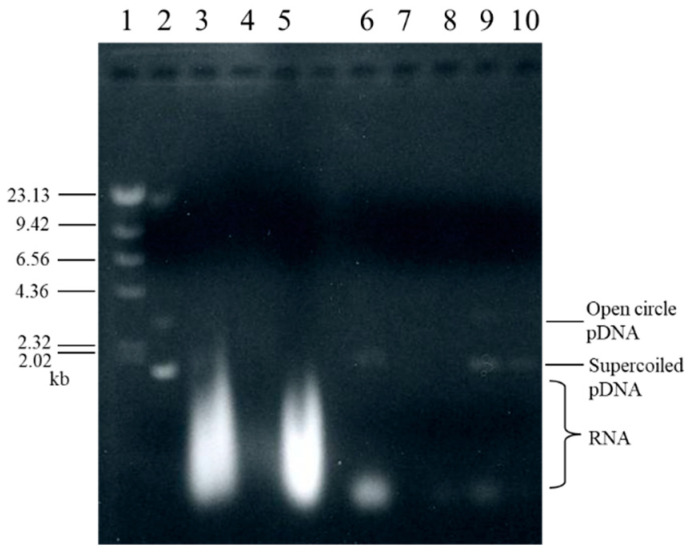
Agarose gel analysis: lane 1, DNA molecular weight standard; lane 2, plasmid DNA; lane 3, *E. coli* lysate; lane 4, supernatant (CaCl_2_ addition); lane 5, sediment (CaCl_2_ addition); lane 6, sediment (ethanol addition); lane 7, filtrate (pH 5); lane 8, permeate (pH 9); lane 9, permeate (pH 10 early phase); lane 10, permeate (pH 10 later phase).

Although plasmid DNA permeates the microfiltration membrane, the ligand *α*-Fe_2_O_3_ particles are captured using the 0.1 μm microfiltration membrane, forming a filter cake. Plasmid DNA can be purified using two-stage microfiltration by adsorbing the plasmid DNA onto the *α*-Fe_2_O_3_ cake to separate impurities and subsequently desorbing from the α-Fe_2_O_3_ cake. Based on the results of the adsorption experiments, the amount of *α*-Fe_2_O_3_ required to adsorb almost 100% of the plasmid DNA in the test solution prepared from *E. coli* was calculated, and adsorption microfiltration experiments were performed. Typical data of the microfiltration experiments of *α*-Fe_2_O_3_ and *α*-Fe_2_O_3_ with plasmid DNA-bound slurries at pH 5 are plotted in Figure 5 in the form of the reciprocal filtration rate (d*θ*/d*v*) against the filtrate volume *v* per unit effective membrane area. For the filtration of the *α*-Fe_2_O_3_ slurry, the plots appeared to be linear according to the Ruth filtration rate equation, expressed as [28]

(2) $$\frac{d\theta }{dv}=\frac{\mu \rho s\alpha_{\mathrm{av}}}{p\left(1-ms\right)}\left(v+v_{m}\right),$$

where *θ* is the filtration time, *μ* is the viscosity of the filtrate, *ρ* is the density of the filtrate, *s* is the mass fraction of the solids in the slurry, *p* is the applied filtration pressure, *v*_m_ is the fictitious filtrate volume per unit effective membrane area, and *m* is the ratio of the mass of the wet cake to the mass of the dry cake. The average specific cake resistance *α*_av_ was calculated from Equation (2) using the slope of the plot. In contrast, for the filtration of *α*-Fe_2_O_3_ with the plasmid DNA-bound slurry, cake formation was significantly affected by particle settling. After the formation of the filter cake, the supernatant fluid permeated the filter cake. During this period, d*θ*/d*v* remained approximately constant. From this constant value (d*θ*/d*v*)_p_, *α*_av_ can be calculated as

(3) $$\alpha_{\mathrm{av}}=\frac{p}{\mu w}\left\{{\left(\frac{d\theta }{dv}\right)}_{p}-{\left(\frac{d\theta }{dv}\right)}_{m}\right\},$$

where *w* is the net solid mass of the entire cake per unit effective membrane area, and (d*θ*/d*v*)_m_ is the reciprocal filtration rate, which is equivalent to the flow resistance of the membrane. It was observed that average specific cake resistance decreased by approximately 1/3 from 3.9 × 10^12^ m/kg to 1.0 × 10^12^ m/kg because of the binding of plasmid DNA to *α*-Fe_2_O_3_. This phenomenon is attributed to the charge neutralization of positively charged *α*-Fe_2_O_3_ by the polyanion plasmid DNA, resulting in floc formation and coarsening. In addition, the filtrate did not contain plasmid DNA, as shown in Figure 6, lane 7.

**Figure 7:**
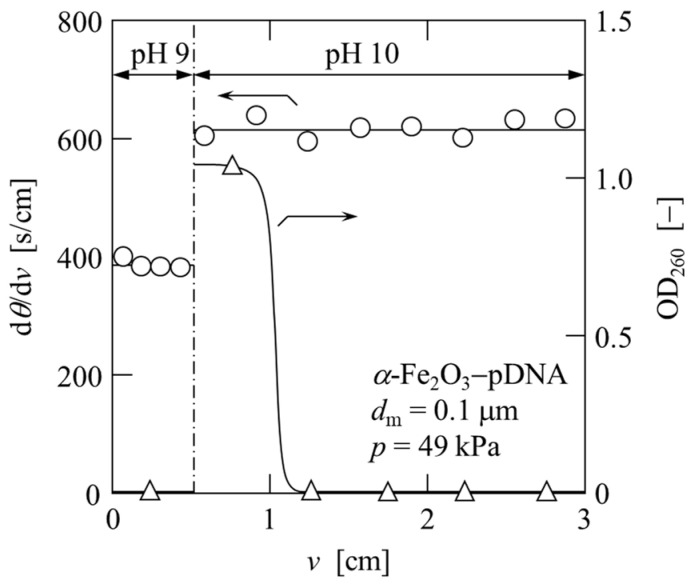
Variation of reciprocal filtration rate and optical density with filtrate volume per unit membrane area in liquid permeation (1 M Tris-HCl buffer pH 9 and 2 M Tris-HCl buffer pH 10).

In Figure 7, d*θ*/d*v* and the optical density at a wavelength of 260 nm (OD_260_) of the permeate are plotted against the permeate volume *v* per unit effective membrane area in the elution process. Upon changing the pH of the permeate from 9 to 10, the value of d*θ*/d*v* changed from 400 s/m to 600 s/cm, indicating a change in the cake structure. A higher pH resulted in a higher average specific cake resistance *α*_av_, as determined from Equation (3). Furthermore, the variation in OD_260_ of the permeate showed that plasmid DNA was eluted in the early phase of the pH 10 liquid permeation (2 M Tris-HCl buffer). This was confirmed using agarose gel analysis, as shown in Figure 6 (lanes 9 and 10). The test solution with OD_260_ = 4.05 was recovered as a solution with OD_260_ = 1.05 using a two-stage affinity microfiltration.

Figure 6 shows the results of the agarose gel electrophoresis of the solutions obtained after each treatment. The solution (lane 6) that was subjected to adsorption filtration after alkaline lysis of *E. coli* (lane 3) and the addition of CaCl_2_ (lanes 4 and 5) were found to contain plasmid DNA and a large amount of low-molecular-weight RNA. Neither plasmid DNA nor RNA was confirmed in the filtrate (lane 7) of the adsorption filtration; therefore, both nucleic acids are assumed to be adsorbed by *α*-Fe_2_O_3_ and exist in the cake on the membrane surface. In the desorption filtration of plasmid DNA, a small amount of low-molecular-weight RNA was confirmed in the permeate of pH 9 (lane 8). Subsequently, plasmid DNA and RNA were confirmed in the initial permeate at pH 10 (lane 9), and only plasmid DNA was confirmed in the subsequent permeate at pH 10 (lane 10). Therefore, highly purified plasmid DNA can be obtained from *E. coli* using a two-stage microfiltration process with adsorption and desorption.

### 3.3. Cell Disruption Properties

**Figure 8:**
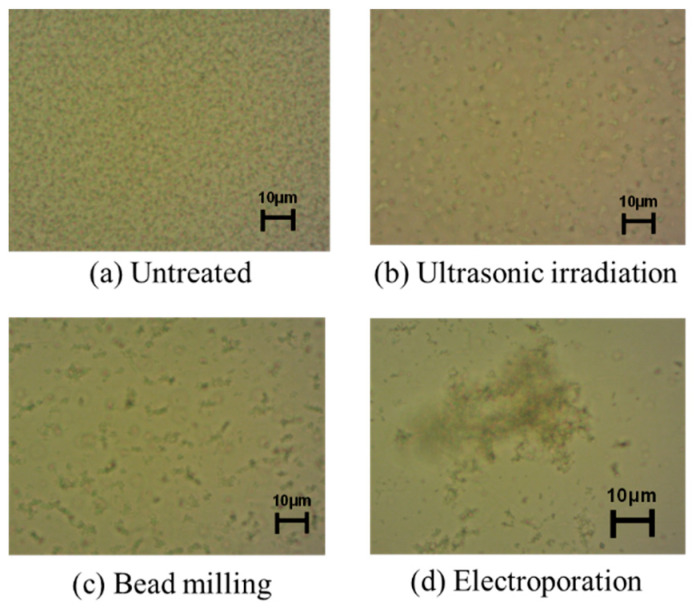
Photomicrographs of *E. coli* suspension treated under various conditions: (**a**) Untreated; (**b**) Ultrasonic irradiation; (**c**) Bead milling; (**d**) Electroporation.

The above study applied the two-stage microfiltration process with adsorption and desorption and was performed on a solution containing plasmid DNA after alkaline lysis of *E. coli*. To establish a safe method that uses the minimum amount of chemicals possible, we investigated a plasmid DNA release method. The release of plasmid DNA from *E. coli* was attempted with ultrasonic irradiation, bead milling, and electroporation. In both methods, plasmid DNA was released from the *E. coli* suspension after treatment. However, long-term ultrasonic irradiation destroys the released plasmid DNA and does not increase the recovery amount, and bead mill disruption cuts the genomic DNA, rendering the subsequent purification difficult. Figure 8 shows micrographs of *E. coli* suspensions treated with each method. Compared with untreated cells, the change after electroporation was remarkable, and large flocs were formed. Biopolymers, such as genomic DNA, polysaccharides, and proteins, were released from *E. coli* using electroporation, and aggregates were formed along with the cells.

**Figure 9:**
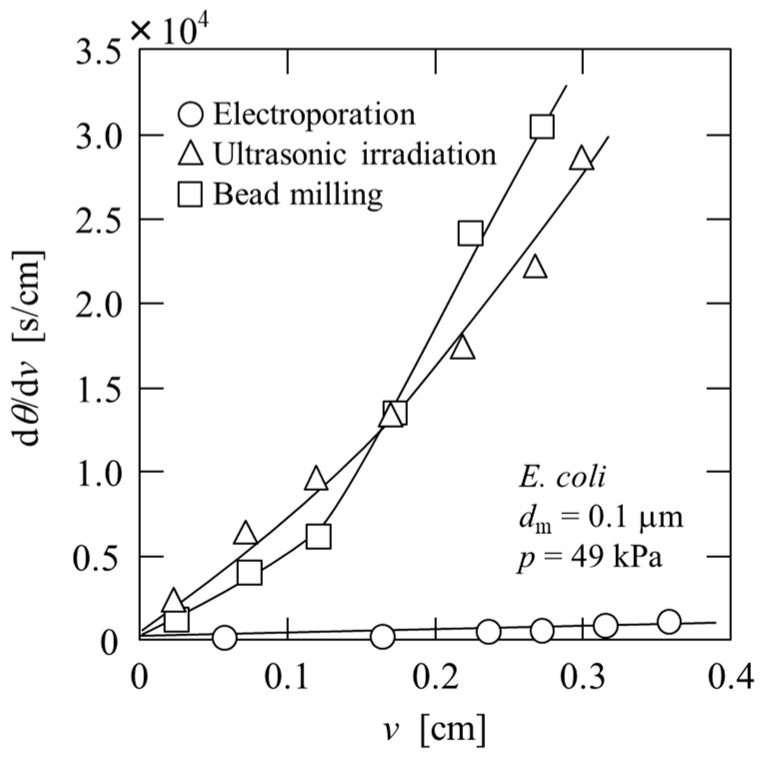
Microfiltration behavior of *E. coli* suspension treated under various conditions.

Microfiltration was performed to remove impurities and obtain a plasmid DNA solution, and the results are plotted in Figure 9 in the form of the reciprocal filtration rate (d*θ*/d*v*) against the filtrate volume *v* per unit effective membrane area. In the case of ultrasonic irradiation and bead milling, the flux decline was significant. In contrast, in the case of electroporation, in which aggregates were formed, the filtration rate was extremely high, confirming the superior separation performance. OD_260_ of the filtrates obtained using ultrasonic irradiation, bead milling, and electroporation were 8.80, 9.85, and 7.08, respectively, and OD_260_/OD_280_ ratios were 1.5, 1.9, and 2.0, respectively. In terms of plasmid DNA extraction, Haberl et al. show that electroextraction leads to a higher concentration of extracted plasmid DNA than alkaline lysis, which is commonly used [22]. This filtrate containing plasmid DNA can be purified using a two-stage microfiltration process using *α*-Fe_2_O_3_, as described above. The recovered solution using a two-stage affinity microfiltration exhibited an OD_260_/OD_280_ ratio of 1.8, indicating a high degree of nucleic acid purification [29]. However, to further improve the degree of purification of plasmid DNA, an operation to remove RNA, such as the addition of CaCl_2_ or degradation with RNase, is required. In addition, attention should be paid to the detection and removal of impurities that do not contribute to the OD_260_/OD_280_ ratio.

## 4. Conclusions

The affinity microfiltration of plasmid DNA using *α*-Fe_2_O_3_ as a ligand was examined. The adsorption and desorption properties of plasmid DNA revealed that *α*-Fe_2_O_3_ particles are suitable ligands. The data from two-stage affinity microfiltration, including both the binding process of plasmid DNA to *α*-Fe_2_O_3_ and the elution process of bound plasmid DNA, demonstrate that this method has immense potential for plasmid DNA purification. However, adsorption and desorption times should be optimized to reduce processing time. In addition, electroporation is effective as an elution method for bacterial cells in the purification process of plasmid DNA. The microfiltration performance was high owing to the formation of aggregates of impurities, including cells. Furthermore, the degree of nucleic acid purification was high. We believe that the results of this study will contribute to the establishment of a purification process suitable for the mass production of pharmaceutical-grade plasmid DNA.

## Data Availability

Not applicable.
